# Association of methionine synthase rs1801394 and methionine synthase reductase rs1805087 polymorphisms with meningioma in adults: A meta-analysis

**DOI:** 10.3892/br.2014.248

**Published:** 2014-03-12

**Authors:** XIAN-TAO ZENG, JUN-TI LU, XIANG-JUN TANG, HONG WENG, JIE LUO

**Affiliations:** 1 Center for Evidence-Based Medicine and Clinical Research, Taihe Hospital, Hubei University of Medicine, Shiyan, Hubei 442000, P.R. China; 2 Department of Neurosurgery, Taihe Hospital, Hubei University of Medicine, Shiyan, Hubei 442000, P.R. China

**Keywords:** methionine synthase, methionine synthase reductase, meningioma, meta-analysis, polymorphism

## Abstract

Several epidemiological studies suggested that methionine synthase (MTRR) rs1801394 and methionine synthase reductase (MTR) rs1805087 polymorphisms may be involved in the risk of meningioma in adults; however, the results from different case-control studies have been inconsistent. Therefore, we performed a meta-analysis to investigate the association of MTRR and MTR polymorphisms with meningioma. PubMed, Web of Knowledge, China National Knowledge Infrastructure and Wanfang databases were searched up to October 30, 2013 and 3 publications, involving 7 case-control studies, were finally included. Following data extraction, a meta-analysis was conducted using Stata 12.0 software. The pooled results based on the fixed effects model demonstrated that the MTRR rs1801394 polymorphism was associated with an increased risk of meningioma [odds ratio (OR)=1.18, 95% confidence interval (CI): 1.05–1.32 for G vs. A; OR=1.41, 95% CI: 1.12–1.77 for GG vs. AA; OR=1.08, 95% CI: 0.94–1.33 for AG vs. AA; OR=1.19, 95% CI: 1.01–1.40 for (AG+GG) vs. AA; and OR=1.32, 95% CI: 1.07–1.63 for GG vs. (AG+AA)]; however, an association between the MTR rs1805087 polymorphism and the risk of meningioma was not identified [OR=0.99, 95% CI: 0.88–1.12 for G vs. A; OR=1.09, 95% CI: 0.80–1.48 for GG vs. AA; OR=0.95, 95% CI: 0.82–1.11 for AG vs. AA; OR=0.97, 95% CI: 0.84–1.13 for (AG+GG) vs. AA; and OR=1.09, 95% CI: 0.80–1.48 for GG vs. (AG+AA)]. Therefore, the currently available evidence suggests that the MTRR rs1801394 polymorphism may increase the risk of meningioma, whereas the MTRR rs1801394 polymorphism is not associated with meningioma.

## Introduction

The majority of meningiomas are benign tumors located intracranially and are often associated with severe and possibly fatal consequences, although they are encapsulated and non-malignant tumors with restricted numbers of genetic aberrations (1). Meningioma is a complex multifactorial disease and is the second most common primary cerebral neoplasm in adults (2). The prevalence of meningioma was reported to be 6/100,000 individuals annually in the United States (3). Environmental factors, such as ionizing radiation (4–7), hormones (8–12), head trauma (13,14) and cell phone use (15,16), as well as genetic factors, contribute to the pathogenesis of meningioma (17–19).

Methionine synthase (MTRR), an enzyme that catalyzes the remethylation of homocysteine to methionine and the concurrent demethylation of 5-methyltetrahydrofolate to tetrahydrofolate, is a vitamin B12-dependent enzyme. Methionine synthase reductase (MTR), which is a reductase catalyzing methyl cobalt amine regeneration, is the cofactor of MTRR and plays a vital role in maintaining MTRR in the folate metabolic process. The MTRR A66G (rs1801394) polymorphism is a well-recognized locus associated with enzyme function and may affect the interaction of MTRR and MTR, participating in tumor formation (20). A common polymorphism in MTR, A2756G (rs1805087), is considered to affect enzyme activity, leading to homocysteine elevation and DNA hypomethylation (21).

Several molecular epidemiological studies have been conducted to investigate the association of MTRR rs1801394 and MTR rs1805087 polymorphisms with meningioma. Yu *et al* (22) performed a meta-analysis based on two publications and indicated that there was no association between the MTR rs1805087 polymorphism and meningioma; however, 3 years later, a primary study reporting an association between MTR rs1805087 and meningioma was published (23). Whether these results are altered by an updated meta-analysis requires clarification. Additionally, an association between the MTRR rs1801394 polymorphism and meningioma has not been confirmed. Therefore, we performed the present meta-analysis to investigate the association of the MTRR rs1801394 and MTR rs1805087 polymorphisms with susceptibility to meningioma. The present meta-analysis was conducted according to the Meta-analysis of Observational Studies in Epidemiology statements (24).

## Materials and methods

### Inclusion criteria

A publication was included if it met the following criteria: i) the patients had been diagnosed with meningioma by magnetic resonance imaging, histological, pathological, or cytological methods; ii) the association of MTRR rs1801394 and/or MTR rs1805087 polymorphism with meningioma susceptibility was investigated; iii) the study design was a case-control study; iv) the number of individual genotypes in the case and control groups was provided or could be calculated using valid information; and v) the publication language was restricted to English or Chinese.

### Search strategy

The PubMed, Web of Knowledge, China National Knowledge Infrastructure and Wanfang databases were searched up to October 30, 2013 using the following key words: (meningioma or meningiomatosis or meningeoma or meningothelioma or durosarcoma or clear cell meningioma or sphenoid ridge meningiomas) and (polymorphism or mutation or saltation or variation or genetic change) and (methionine synthase or MTRR or methionine synthase reductase or MTR). The bibliographies of the included articles were hand-searched to identify relevant meta-analyses and recent reviews.

### Data extraction

Two investigators independently performed data extraction according to the pre-specified inclusion criteria. Any disagreements were resolved by consulting a third investigator. The following information were extracted: surname of the first author, publication year, country, ethnicity, source of controls, sample size, genotyping method, genotyping distribution in the case and control groups and Hardy-Weinberg equilibrium (HWE) for controls.

### Statistical analysis

The odds ratios (ORs) and corresponding 95% confidence intervals (CIs) were calculated for five genetic models in both MTRR rs1801394 and MTR rs1805087 polymorphisms: G vs. A, GG vs. AA, AG vs. AA, (AG+GG) vs. AA and GG vs. (AG+AA). The heterogeneity among the included case-control studies was assessed using I^2^ statistics (25) and the fixed effects model was used if I^2^≤50%, which indicated no significant heterogeneity among the included articles (26); otherwise, the random effects model was applied. A stratified analysis based on ethnicity, HWE of controls and source of controls was also conducted. Publication bias was assessed by Egger’s linear regression test (27). Additionally, we performed sensitivity analyses by excluding each study individually and recalculated the ORs and corresponding 95% CIs. All the data were calculated using Stata 12.0 software (StataCorp, College Station, TX, USA). The statistical tests used a two-sided P-value, with the level of significance set at <0.05.

## Results

### Study selection and characteristics

Our search strategy, initially identified 12 publications. Following removal of duplicates and non-relevant articles, 3 publications with 7 case-control studies, including a total of 1,521 meningioma patients and 1,523 healthy controls for MTR rs1805087 polymorphism and 1,231 meningioma patients and 1,237 healthy controls for MTRR rs1801394 polymorphism, were finally included (23,28,29).

Three articles involving 7 case-control studies focused on MTR rs1805087 polymorphism (23,28,29) and 2 articles involving 6 case-control studies focused on MTRR rs1801394 polymorphism (23,28). Only one study (23) focused on an Asian population and the HWE of the controls was <0.05; the remaining studies focused on Caucasian populations. The genotyping method included polymerase chain reaction (PCR) (29), PCR-restriction fragment length polymorphism (PCR-RFLP) (23) and Illumina GoldenGate arrays (Illumina, San Diego, CA, USA) (28). The characteristics of the included studies are summarized in Table I.

### Meta-analysis

The meta-analysis results of the overall population based on the fixed effects model are presented in Table II. The MTRR rs1801394 polymorphism was significantly associated with meningioma in four genetic models [OR=1.18, 95% CI: 1.05–1.32 for G vs. A; OR=1.41, 95% CI: 1.12–1.77 for GG vs. AA; OR=1.32, 95% CI: 1.07–1.63 for GG vs. (AG+AA); and OR=1.19, 95% CI: 1.01–1.40 for (AG+GG) vs. AA; except AG vs. AA (OR=1.12, 95% CI: 0.94–1.33)]. When stratified by ethnicity, a significant difference was only identified in two genetic models for Caucasian populations (OR=1.19, 95% CI: 1.02–1.40 for G vs. A; and OR=1.40, 95% CI: 1.01–1.94 for GG vs. AA) and only two genetic models exhibited a significant difference for Asian populations [OR=1.41, 95% CI: 1.02–1.96 for GG vs. AA; and OR=1.39, 95% CI: 1.03–1.87 for GG vs. (AG+AA)]. The MTR rs1805087 polymorphism exhibited no significant association with meningioma in the five genetic models. We also did not observe any statistically significant difference in the subgroups based on ethnicity, HWE of controls and source of controls.

The sensitivity analysis was performed by removing each of the included studies of the meta-analysis and the results demonstrated that the ORs were not significantly altered, suggesting that the results were statistically robust and credible.

### Publication bias

The publication bias was assessed with the Egger’s linear regression test (27) and there was no evidence of publication bias in this meta-analysis of any genetic model. The detailed data are presented in Table II.

## Discussion

Folate metabolism gene polymorphisms have been implicated in the pathogenesis of meningioma and numerous studies have evaluated the association between the two. Evidence from a previous meta-analysis (30) demonstrated that the methylenetetrahydrofolate reductase C677T polymorphism may modify the risk of meningioma, whereas another meta-analysis (22) reported that the MTR rs1805087 polymorphism exhibited no significant association with meningioma. As regards MTR rs1805087 polymorphism, a large-sample size case-control study was published (23), which also focused on MTRR rs1801394. However, the association of MTRR rs1801394 polymorphism with meningioma remains unclear. Therefore, we conducted the present meta-analysis to investigate the association of MTRR rs1801394 polymorphism with meningioma and provide updated information on the association of MTR rs1805087 polymorphism with meningioma.

The overall result of the present meta-analysis, based on 6 case-control studies (23,28) focused on MTRR rs1801394 polymorphism, revealed a statistically significant difference in four genetic models (Table II). The pooled results indicated an association between the MTRR rs1801394 polymorphism and susceptibility to meningioma. However, in the subgroup analyses, no significant difference was observed. A recently published case-control study, involving a total of 1,200 individuals, which was based on an Asian population and reported an HWE of <0.05 for the control group, was included in our meta-analysis (23). The results of that study revealed a marginally significant association between MTRR rs1801394 polymorphism and meningioma in the GG vs. AA and GG vs. (AG+AA) models. However, when stratifying by the World Health Organization grade of meningioma, no association was observed. The remaining studies were all focused on Caucasian populations and their results revealed a marginally significant association between the MTRR rs1801394 polymorphism and meningioma in the GG vs. AA and GG vs. (AG+AA) geentic models. Therefore, the results should be interpreted with caution.

The aggregated results of our study, based on 7 case-control studies (23,28,29) on MTR rs1805087 polymorphism, revealed no significant differences between MTR rs1805087 polymorphism and meningioma in all the genetic models and subgroup analyses after including the large-sample size study (23). Compared to the previous meta-analysis (22) focusing on Caucasian populations, which reported no association between the MTR rs1805087 polymorphism and meningioma, our meta-analysis demonstrated that there was no association between the MTR rs1805087 polymorphism and meningioma in an Asian population. However, this result was only based on a case-control study focusing on an Asian population.

There were certain limitations to our meta-analysis. First, the sample size was the most important limiting factor, as the included studies were relatively small. Second, ethnicity played an important role, as we found that the MTRR rs1801394 polymorphism may increase the susceptibility to meningioma in four genetic models, except the AG vs. AA model, whereas we observed that only two genetic models exhibited an association between the MTRR rs1801394 polymorphism and meningioma in Caucasian and Asian populations. This may be the most important confounding factor in our meta-analysis and the results must be interpreted with caution. Third, due to the limited number of included studies, we were unable to perform subgroup analyses according to the classification of meningioma between single-nucleotide polymorphisms and meningioma to assess the dose-response. Finally, our meta-analysis was based on unadjusted estimates, similar to all meta-analyses of polymorphisms, due to the lack of original data from the included studies. Therefore, the investigation of gene-gene and/or gene-environment interactions was restricted.

In conclusion, the present meta-analysis, based on 1,521 meningioma patients and 1,523 healthy controls for MTR rs1805087 polymorphism and 1,231 meningioma patients and 1,237 healthy controls for MTRR rs1801394 polymorphism, demonstrated that the MTRR rs1801394 polymorphism is associated with an increased risk of meningioma, whereas the MTR rs1805087 polymorphism is not associated with meningioma. However, a definitive conclusion cannot be reached due to the limitations of our meta-analysis. Therefore, further large-sample size studies are required to determine the true association between these two single-nucleotide polymorphisms and meningioma in different ethnicities.

## Figures and Tables

**Table I:** Characteristics of the included studies.

A, Methionine synthase reductase A2756G (rs1805087)												

Author (year)	Country (ethnicity)	Cases/controls	Source of controls	Cases			Genotyping method	Controls			P-value for HWE	(Refs.)
	
				AA	AG	GG		AA	AG	GG		
Semmler *et al* (2008)	Germany (Caucasian)	290/287	HB	197	81	12	PCR	184	92	11	0.91	(29)
Zhang *et al* (2013)	China (Asian)	600/600	PB	347	198	55	PCR-RFLP	361	190	49	0.001	(23)
Bethke *et al* (2008)	Denmark (Caucasian)	110/113	PB	73	33	4	Illumina	70	40	3	0.33	(28)
Bethke *et al* (2008)	UK-North (Caucasian)	174/174	PB	113	54	7	Illumina	106	60	8	0.89	(28)
Bethke *et al* (2008)	UK-Southeast (Caucasian)	121/123	PB	77	39	5	Illumina	75	42	6	0.97	(28)
Bethke *et al* (2008)	Finland (Caucasian)	77/77	PB	50	24	3	Illumina	56	17	4	0.099	(28)
Bethke *et al* (2008)	Sweden (Caucasian)	149/149	PB	98	45	6	Illumina	94	51	4	0.34	(28)

B, Methionine synthase A66G (rs1801394)												

Author (year)	Country (ethnicity)	Cases/controls	Source of controls	Cases			Genotyping method	Controls			P-value for HWE	(Refs.)
	
				AA	AG	GG		AA	AG	GG		

Zhang *et al* (2013)	China (Asian)	600/600	PB	209	269	122	PCR-RFLP	225	282	93	0.77	(23)
Bethke *et al* (2008)	Denmark (Caucasian)	110/113	PB	41	47	22	Illumina	40	55	18	0.9	(28)
Bethke *et al* (2008)	UK-North (Caucasian)	174/175	PB	54	83	37	Illumina	74	78	23	0.73	(28)
Bethke *et al* (2008)	UK-Southeast (Caucasian)	121/123	PB	41	57	23	Illumina	39	59	25	0.76	(28)
Bethke *et al* (2008)	Finland (Caucasian)	77/77	PB	26	37	14	Illumina	30	33	14	0.36	(28)
Bethke *et al* (2008)	Sweden (Caucasian)	149/149	PB	39	84	26	Illumina	53	74	22	0.64	(28)

HWE, Hardy-Weinberg equilibrium; HB, hospital-based; PB, population-based; PCR-RFLP, polymerase chain reaction-restriction fragment length polymorphism.

**Table II:** Results of the overall and subgroup analyses.

A, Methionine synthase reductase A2756G (rs1805087)											

		G vs. A		GG vs. AA		AG vs. AA		(AG+GG) vs. AA		GG vs. (AG+AA)	
						
Variables	N	OR (95% CI)	I^2^ (%)	OR (95% CI)	I^2^ (%)	OR (95% CI)	I^2^ (%)	OR (95% CI)	I^2^ (%)	OR (95% CI)	I^2^ (%)
Overall	7	0.99 (0.88–1.12)	0	1.09 (0.80–1.48)	0	0.95 (0.82–1.11)	0	0.97 (0.84–1.13)	0	1.09 (0.80–1.48)	0
Egger’s test		P=0.35		P=0.36		P=0.73		P=0.53		P=0.57	
Ethnicity											
Caucasian	6	0.92 (0.78–1.09)	0	0.99 (0.62–1.59)	0	0.88 (0.72–1.07)	0	0.89 (0.74–1.08)	0	1.03 (0.65–1.65)	0
Asian	1	1.09 (0.91–1.32)	NA	1.17 (0.77–1.76)	NA	1.08 (0.85–1.39)	NA	1.10 (0.87–1.39)	NA	1.13 (0.76–1.70)	NA
Source of controls											
PB	6	1.02 (0.89–1.16)	0	1.10 (0.79–1.54)	0	0.99 (0.83–1.17)	0	1.00 (0.85–1.18)	0	1.09 (0.79–1.51)	0
HB	1	0.89 (0.66–1.20)	NA	1.02 (0.44–2.37)	NA	0.82 (0.57–1.18)	NA	0.84 (0.60–1.19)	NA	1.08 (0.47–2.50)	NA
HWE											
>0.05	6	0.92 (0.78–1.09)	0	0.99 (0.62–1.59)	0	0.88 (0.72–1.07)	0	0.89 (0.74–1.08)	0	1.03 (0.65–1.65)	0
<0.05	1	1.09 (0.91–1.32)	NA	1.17 (0.77–1.76)	NA	1.08 (0.85–1.39)	NA	1.10 (0.87–1.39)	NA	1.13 (0.76–1.70)	NA

B, Methionine synthase A66G (rs1801394)											

		G vs. A		GG vs. AA		AG vs. AA		(AG+GG) vs. AA		GG vs. (AG+AA)	
						
Variables	N	OR (95% CI)	I^2^ (%)	OR (95% CI)	I^2^ (%)	OR (95% CI)	I^2^ (%)	OR (95% CI)	I^2^ (%)	OR (95% CI)	I^2^ (%)

Overall	6	1.18 (1.05–1.32)	0	1.41 (1.12–1.77)	0	1.12 (0.94–1.33)	0	1.19 (1.01–1.40)	3.5	1.32 (1.07–1.63)	0
Egger’s test		P=0.72		P=0.71		P=0.63		P=0.83		P=0.39	
Ethnicity											
Caucasian	5	1.19 (1.02–1.40)	15.3	1.40 (1.01–1.94)	3.8	1.21 (0.94–1.54)	0	1.26 (1.00–1.59)	15.4	1.26 (0.94–1.68)	0
Asian	1	1.17 (0.99–1.37)	NA	1.41 (1.02–1.96)	NA	1.03 (0.80–1.32)	NA	1.12 (0.89–1.42)	NA	1.39 (1.03–1.87)	NA

OR, odds ratio; CI, confidence interval; NA, not available; PB, population-based; HB, hospital-based; HWE, Hardy-Weinberg equilibrium.
